# AKT1^low^ quiescent cancer cells persist after neoadjuvant chemotherapy in triple negative breast cancer

**DOI:** 10.1186/s13058-017-0877-7

**Published:** 2017-08-01

**Authors:** Sheheryar Kabraji, Xavier Solé, Ying Huang, Clyde Bango, Michaela Bowden, Aditya Bardia, Dennis Sgroi, Massimo Loda, Sridhar Ramaswamy

**Affiliations:** 1 Massachusetts General Hospital Cancer Center, Richard B. Simches Research Building, 185 Cambridge Street, Boston, MA 02114 USA; 2 Harvard Medical School, Boston, MA USA; 3 Dana-Farber Cancer Institute, Boston, MA USA; 4 Broad Institute of Harvard & MIT, Cambridge, MA USA; 5 Harvard Stem Cell Institute, Cambridge, MA USA; 6 Harvard-Ludwig Center for Cancer Research, Boston, MA USA; 7 Present address: Cancer Prevention and Control Program, Catalan Institute of Oncology-IDIBELL, Barcelona, Spain

**Keywords:** AKT1^low^ quiescent cancer cells, Chemotherapy resistance, Quantitative immunofluorescence microscopy

## Abstract

**Background:**

Absence of pathologic complete response (pCR) to neoadjuvant chemotherapy (NACT) correlates with poor long-term survival in patients with triple negative breast cancer (TNBC). These incomplete treatment responses are likely determined by mechanisms that enable cancer cells to resist being killed. However, the detailed characterization of a drug-resistant cancer cell state in residual TNBC tissue after NACT has remained elusive. AKT1^low^ quiescent cancer cells (QCCs) are a quiescent, epigenetically plastic, and chemotherapy-resistant subpopulation initially identified in experimental cancer models. Here, we asked whether QCCs exist in primary tumors from patients with TNBC and persist after treatment with NACT.

**Methods:**

We obtained pre-treatment biopsy, post-treatment mastectomy, and metastatic specimens from a retrospective cohort of TNBC patients treated with NACT at Massachusetts General Hospital (*n* = 25). Using quantitative automated immunofluorescence microscopy, QCCs were identified as AKT^low^/H3K9me2^low^/HES1^high^ cancer cells using prespecified immunofluorescence intensity thresholds. QCCs were represented in 2D and 3D digital tumor maps and QCC percentage (QCC-P) and QCC cluster index (QCC-CI) were determined for each sample.

**Results:**

We showed that QCCs exist as non-random and heterogeneously distributed clusters within primary breast tumors. In addition, these QCC clusters persist after treatment with multi-agent, multi-cycle, neoadjuvant chemotherapy in both residual primary tumors and nodal and distant metastases in patients with triple negative breast cancer.

**Conclusions:**

These first-in-human data potentially qualify AKT1^low^ quiescent cancer cells as a non-genetic cell state that persists after neoadjuvant chemotherapy in triple negative breast cancer patients and warrants further study.

**Electronic supplementary material:**

The online version of this article (doi:10.1186/s13058-017-0877-7) contains supplementary material, which is available to authorized users.

## Background

Triple negative breast cancer (TNBC) is more commonly diagnosed in younger and African-American women, is associated with worse recurrence-free survival and worse overall survival, and has fewer effective treatment options, compared to hormone receptor-positive or human epidermal growth factor receptor 2 (HER2)-positive breast cancer [1, 2]. For patients with localized TNBC, neoadjuvant chemotherapy (NACT) is primarily used to increase the success of breast conservation therapy [3]. This neoadjuvant approach also offers an attractive paradigm for evaluating biomarkers of resistance to chemotherapy, since pre-treatment tumor biopsies can be compared to post-treatment mastectomy specimens and pathologic complete response (pCR) to NACT correlates with better clinical outcomes in TNBC [4]. While pCR rates reported in clinical trials vary depending on the definition used, tumor type, and specific neoadjuvant treatment, poor pathologic response despite NACT occurs in 40–60% of patients with TNBC [5]. Absence of pCR after neoadjuvant chemotherapy is presumably determined by mechanisms that enable tumor cells to resist being killed. A small number of tumor-specific genetic alterations have been recently described in localized TNBC after NACT (e.g. *TP53, MCL1, MYC*), although actionable targets remain infrequent [6]. Alternative mechanisms that give rise to drug-resistant cell states such as quiescence or altered epigenetic profile have also been postulated, although the detailed characterization of a putative quiescent, drug-resistant, cancer cell state in residual TNBC after NACT remains elusive [6–9].

We previously reported that MCF7 (estrogen receptor positive (ER+)) and MDA-MB-231 (triple negative) breast cancer cell lines (among other tumor types) are capable of dividing asymmetrically to form quiescent, AKT1^low^ cancer cells (QCCs) in response to decreased integrin-β1 (ITGB1) signaling from the microenvironment both in vitro and in vivo [10, 11]. We have previously shown that these AKT1^low^ slow proliferators also express other markers of quiescence in being ROS^low^, MKI67^low^, MCM2^low^, H3K9me2^low^ and HES1^high^ [10, 11]; QCCs are intrinsically resistant to cytotoxic chemotherapy and also elaborate CD63+ exosomes that increase the fitness of surrounding, more rapidly cycling, cancer cells [12]. Moreover, we have found that QCCs exhibit many properties ascribed to cancer stem cells, including an increased tumor-initiating capacity and epigenetic plasticity that contributes to cancer cell heterogeneity [13]. Together, these experimental data suggest that AKT1^low^ quiescent cancer cells may play a role in resistance to cytotoxic therapy and tumor progression.

We previously identified QCCs in a small number of primary breast tumors of different subtypes (ER+, triple negative, HER2+) before and after neoadjuvant chemotherapy with indirect immunofluorescence confocal microscopy (*n* = 5) [11]. While this approach is used routinely to identify protein-based biomarkers in formalin-fixed paraffin-embedded (FFPE) human tumors, it is constrained by limited multiplexing, high background, restricted sampling of tumor areas and subjective determination of positive/negative thresholds [14]. In contrast, quantitative immunofluorescence, using a tyramide-signal-amplified (TSA) staining protocol coupled to multispectral imaging and automated tissue segmentation, allows for the objective detection of multiple intracellular protein targets within defined regions of interest and enables accurate and reproducible assessment of individual cell markers and cell states in a wide variety of human tumor samples [15–18]. Here, we used TSA multiplexed immunofluorescence staining coupled to automated multispectral imaging and computational analysis to identify AKT1^low^ (AKT^low^, H3K9me2^low^, HES1^high^) QCCs in tumors from patients with TNBC. We tested the hypothesis that QCCs persist in primary and metastatic TNBC breast tumors after NACT.

## Methods

### Patient cohort and samples

**Fig. 1:**
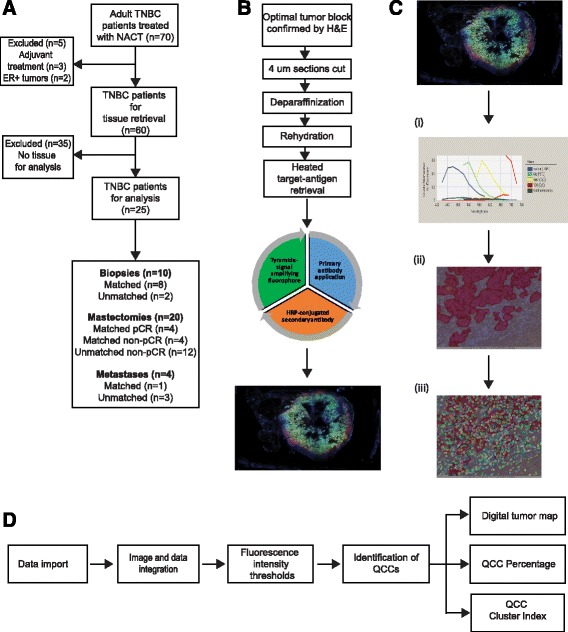
Detailed workflow for AKT1^low^ quiescent cancer cell (*QCC*) identification using tyramide signal amplified immunofluorescence (TSA-IF) automated microscopy coupled with computational image analysis. **a** Consolidated standards of reporting trials (CONSORT) diagram for QCC analysis of tissues from a cohort of patients with triple negative breast cancer (*TNBC*). **b** Scheme demonstrating steps in TSA-IF staining of tumor sections. **c** Scheme demonstrating steps for automated image acquisition using the Vectra platform (Perkin Elmer) and inForm software. Individual images at × 20 undergo spectral isolation for each fluorescent marker (4',6-diamidino-2-phenylindole (*DAPI*), pan-AKT, H3K9me2, HES1) (*i*) to allow for generation of a tissue mask that segregates tumor and stroma (*ii*) followed by a cellular segmentation (*iii*) that allows for extraction of fluorescence intensities for each cellular segment e.g. nucleus, cytoplasm. **d** Scheme demonstrating establishment of fluorescence intensity thresholds for each marker (pan-AKT, H3K9me2, HES1) and identification of QCCs based on pre-established thresholds to allow for generation of digital tumor maps and determination of QCC percentage and QCC cluster index. *NACT* neoadjuvant chemotherapy, *ER+* estrogen receptor positive, *HRP* horseradish peroxidase

We identified a retrospective cohort of patients with localized TNBC at Massachusetts General Hospital, who underwent neoadjuvant chemotherapy between 2010 and 2014. Tissue samples from 25 patients (10 biopsy samples, 20 mastectomy samples, 4 metastatic samples) were retrieved under an Institutional Review Board (IRB)-approved discarded-tissue protocol (2009P20032) as summarized in the consolidated standards of reporting trials (CONSORT) diagram (Fig. 1a); of these 25 patients, 8 patients had matched biopsy and mastectomy samples available for analysis, and of these 8 matched patients, 4 had tumors that underwent pCR, and the remaining 4 patients had primary tumors with residual disease after NACT, which were evaluated for QCCs. One patient with a paired biopsy and mastectomy sample with residual disease also had a metastatic tissue sample available for analysis. An additional 2 unmatched biopsies, 12 unmatched mastectomies and 3 unmatched metastases were also available for QCC analysis. Throughout this report, the term sample refers to a single 4-μm tissue section from a single patient tumor.

### Pathologic assessment of patient samples

Clinical and pathological information (Fig. 4a) was extracted from the medical record. Residual cancer burden was graded, using standard criteria [19] for assessment, on a representative H&E-stained section from each post-treatment mastectomy block, by our study pathologist (DS), who was blinded to QCC results.

### Selection of QCC markers

We have previously shown that QCCs can be reliably identified in vitro and in FFPE human tissues using the antibody profile: pan-AKT^low^, H3K9me2^low^, HES1^high^ [11]. In choosing markers with which to identify QCCs in FFPE patient tissues, we were limited by existing immunofluorescence multiplexing bandwidth to three markers. Therefore, we selected the minimum set of markers that represented canonical features of QCCs, such as quiescence, which we had previously described in vitro [10–12]. To maximize specificity, we opted for at least one marker that was upregulated and another that was downregulated in QCCs.

### Tyramide-signal-amplifying immunofluorescence (TSA-IF) labeling

We undertook TSA-IF labelling of FFPE patient tissue sections as previously described and summarized in Fig. 1 [18]. Primary antibodies were applied iteratively followed by secondary antibody conjugated to horseradish peroxidase (HRP), after which TSA-conjugated fluorophores were applied. Primary antibodies used were, iteratively, H3K9me2 (1:150, abcam ab1220), pan-AKT (1:3000, Cell Signaling 4961, C67E7), and HES1 (1:3000, EMD Millipore AB5702). Primary antibodies had been previously independently validated for target specificity [20–22]. This was followed by a secondary antibody conjugated to HRP (SuperPicture Polymer Detection Kit, Life Technologies), and TSA conjugated to a fluorophore (fluorescein isothiocyanate (FITC), cyanine (CY)3, CY5, respectively, at 1:50 dilution, Perkin Elmer), with intervening wash steps. An alternative iteration of primary antibody application (pan-AKT ➔ H3K9me2 ➔ HES1) was used to confirm target specificity and absence of primary antibody cross-reactivity (Additional file 1: S2). We applied 4',6-diamidino-2-phenylindole (DAPI) as a nuclear counterstain. Staining was completed in batches of 10 and slides were mounted using ProLong Diamond Antifade Mountant without DAPI (ThermoFisher Scientific).

To examine the effects of batch-to-batch staining variability, we used 4 untreated primary breast tumors (control tumors 1–4, Fig. 3b). Control tumors 1 and 2 underwent contiguous (4 μm apart) sequential sectioning (*n* = 2) and sections were stained in different batches. Control tumor 4 underwent contiguous sequential sectioning (*n* = 3) and all sections were stained in the same batch. Control tumor 3 underwent non-contiguous (>4 μm apart) sequential sectioning (*n* = 5) and each section was stained in a different batch containing patient samples, to control for inter-batch variation.

### Confocal microscopy

**Fig. 2:**
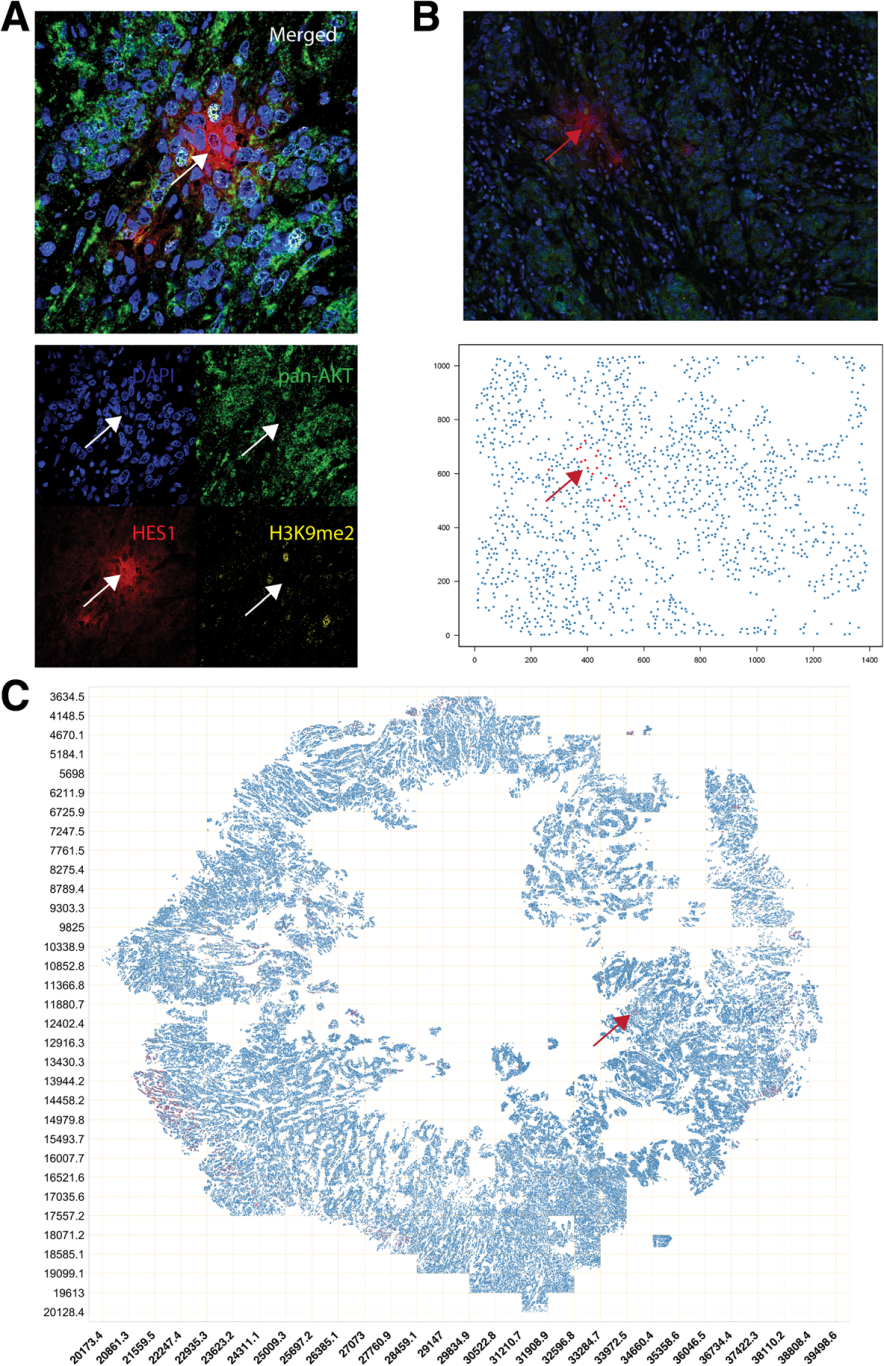
AKT1^low^ quiescent cancer cells (QCCs) are found in primary breast tumor tissue using quantitative immunofluorescence microscopy. **a** *White arrow* points to an example of a QCC cluster from control tumor 3, section 17 by confocal microscopy at × 60; *upper panel* shows merged image and *lower pane*l shows single color images. **b** *Upper panel* shows the same QCC cluster as in **a** (*red arrow*) as × 20 composite image (pan-AKT (*green*), H3K9me2 (*yellow*), and HES1 (*red*)). In the *lower panel*, the area shown in the × 20 image is represented as a digital tumor map identifying the same QCCs (*red arrow*) shown in **a** with high fidelity. **c** An example of a whole-section digital tumor map for tumor 3, section 17, where QCCs are shown by *red dots* and other cancer cells by *blue dots*. Image coordinates are listed on each *axis. Red arrow* points to the same QCC cluster shown in **a** and **b**

To provide a high-resolution example image of a QCC, section 17 from control tumor 3 was imaged using a Nikon Ti confocal microscope at × 60 magnification (Fig. 2a). Merged and single-color images were labelled using Image J [23].

### Spectral imaging and multispectral analysis

Matched H&E-stained slides from each tissue block  were reviewed by a collaborating pathologist (YH) to confirm the presence of tumor cells in each tissue block. Multispectral imaging of sections was undertaken as previously described [18]. TSA-IF-stained slides were scanned using the Vectra slide scanner (V2.0.8, PerkinElmer) with appropriate fluorescent filters. A scanning protocol was created for multispectral imaging and applied to all slides uniformly (Fig. 1c). Regions of interest were manually selected within the Vectra protocol using low-power field previews of the whole slides as reference and scanned to generate a multispectral image at × 20 magnification. Those images with <1% tumor component or >70% technical artifacts (e.g. significant tissue folding, air bubbles, or loss of tissue) were excluded. Single-stained (individual marker with specific fluorophore e.g. only pan-AKT with FITC) TNBC primary tumor sections and blank control slides were used to build a spectral library for each batch (Fig. 1c). InForm V.2.1.1 software (CRi) was used to analyze the spectral images. An InForm tissue and cell segmentation algorithm was developed by selecting representative areas from a training set of 15–20 images, to classify tissue into “tumor” (tumor epithelium) and “stroma” (tumor adjacent tissue) categories. Nuclear segmentation was based on the DAPI signal, with the cytoplasm estimated up to 6 pixels outer distance to nucleus. Tissue classification and cell segmentation were manually reviewed by our study pathologist (YH) to ensure appropriate classification.

### Computational and statistical methods

Raw fluorescence intensity data processing, analysis, and graphical representation of the resulting digital tumor maps were done using R statistical computing software (R Core Team (2015), R Foundation for Statistical Computing, Vienna, Austria).

QCC percentage (QCC-P) for the biopsy, mastectomy and metastasis samples was determined from a single tissue section taken from a single tumor. For groups (biopsy samples or mastectomy samples) mean ± SD values are reported. The difference in mean QCC-P between the pre-treatment biopsy group and the post-treatment mastectomy group was tested using the unpaired *t* test with two-sided *p* < 0.05 as the critical value. The difference in mean QCC cluster index (QCC-CI) between the biopsy group and the mastectomy group was tested using the unpaired *t* test with two-sided *p* < 0.05 as the critical value. Correlation between QCC-P, QCC-I, tumor cellularity was estimated using Pearson’s correlation coefficient.

We undertook a post-hoc power calculation based on our sample size of 10 biopsy samples and 16 mastectomy samples. The difference in mean QCC-P between the biopsy group and the mastectomy group was 0.567 ± 0.25 and, assuming a type 1 error rate of 0.05, the power of this study to detect this difference was 0.73.

### Determination of immunofluorescence intensity thresholds

The average pixel intensity data for the nuclear compartment were used for the H3K9me2 signal, while average pixel intensity data for the cytoplasm compartment were used for the HES1 and AKT. Untreated primary breast tumors (*n* = 4) were designated “control tumors 1–4” and used as a training set to determine fluorescence intensity thresholds.

Using this control tumor training set, we first asked if an absolute threshold of fluorescence intensity could be used for each marker (pan-AKT, H3K9me2, HES1) to define QCCs. We plotted the proportion of cells against fluorescence intensity from two sequential sections stained simultaneously (control tumor 4, Additional files 2: S1A and S1B, respectively). The proportion of cells that expressed each QCC marker (e.g. HES1) at a given fluorescence intensity was not the same in sequential sections (Additional files 2: S1A and S1B, respectively). This suggested that absolute fluorescence intensity thresholds for determining QCCs would not be reproducible across different tumor samples.

Using the control tumor training set, we then asked if a relative threshold could be used to determine fluorescence intensity. We chose three relative thresholds: 25%, 33%, and 50%. For example, at the 25% threshold, cells were called QCCs if they fell into the 75^th^ percentile of fluorescence intensity for HES1 and the 25^th^ percentile of fluorescence intensity for pan-AKT and H3K9me2, respectively, in that section. We applied these three thresholds to our training set control samples (control tumors 1–4, Additional file 2: S1C-E) We found a proportional increase in mean QCC-P and QCC density (QCC-D) (QCC-P per × 20 field of view) with increasing threshold, suggesting no natural cutoff could be detected at the thresholds tested in these control tumors.

Together, these data suggest that there was a continuous expression of each marker (pan-AKT, H3K9me2, HES1) with the absence of a natural cutoff for identifying QCCs. The lack of a natural cutoff has been previously reported for other markers associated with cell proliferation, such as Ki67 [24]. In order to balance specificity and sensitivity, QCCs were defined as those cancer cells expressing the top 25% of fluorescence intensity values for HES1 (HES1^high^) and the bottom 25% of fluorescence intensity values for both pan-AKT (AKT^low^) and H3K9me2 (H3K9me2^low^) using this microscopy approach.

Digital tumor maps were generated by de-convoluting cell geographical coordinates from the relative position of each × 20 magnified image from each section. A single cell Cartesian coordinate system was created for each section and used to accurately represent tumor cell geography in a single map. For improved visualization of QCCs in digital tumor maps (red dots) red hue was maximized using Adobe Photoshop CC 2017 software.

### Definition of QCC-P and QCC-D

We defined QCC-P as the proportion of QCCs in the total cancer cell population per section. We also defined QCC-D as the QCC-P per × 20 field of view (FOV). QCC-P for the biopsy, mastectomy and metastasis samples was determined from a single tissue section taken from a single patient.

### Definition of QCC-CI

The QCC-CI was defined as the proportion of QCCs among the 100 nearest tumor cells for each QCC in a section. Cells with fewer than 100 neighbors in a reasonable proximity (a window of +/- 500 coordinate units in each axis) were discarded. The final QCC-CI for each section was computed by averaging the cluster index for each QCC in that section. Class-label permutation was used to computationally determine the statistical significance of these results. For each sample, 1000 random sets of *k* cells, where *k* is the number of QCCs in the sample, were selected and for each one of these *k* sets of cells a QCC-CI was computed. Once we collected all 1000 permutation-based QCC-CI for a sample, empirical *p* values were obtained by comparing them to the score for that sample.

## Results

In order to test the hypothesis that QCCs persist after NACT in patients with TNBC, we first used a training set of primary breast tumors (control tumors 1–4) to develop a QCC identification platform involving TSA-IF labeling of FFPE tissue sections, spectral imaging, and computational analysis as summarized in Fig. 1.

### QCCs are distributed heterogeneously within primary breast tumors

**Fig. 3:**
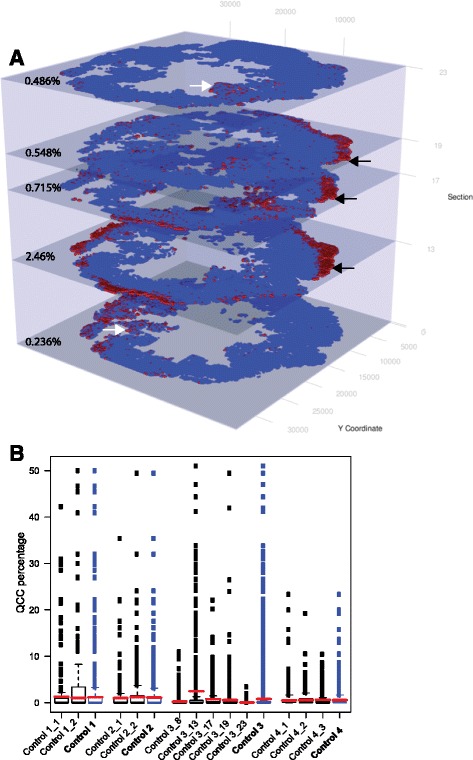
AKT1^low^ quiescent cancer cells (*QCC*) are heterogeneously and non-randomly distributed throughout primary breast tumors. **a** Control tumor 3, sections 8–23 arrayed sequentially as a 3D stack of digital tumor maps showing QCCs (*red dots*) and other tumor cells (*blue dots*). Stroma, areas of necrosis or areas of poor image quality are excluded (*gray areas*). QCC percentage (QCC-P) for each section is shown on the *left* of each plane. QCCs are found in the periphery of some tumor sections but not others (*black arrows* vs. *white arrows*). **b** Summary of QCC-P for each section of control samples 1–4 (*red lines*) with QCC-density (QCC-P for each × 20 field of view) represented by *box and whisker plot* for each section. Summary QCC-P and QCC density for each whole tumor sample (mean of all available sections from that tumor) is indicated by *bold labels* (e.g. ***Control 3***)

Using the QCC identification platform, we were able to identify and represent AKT1^low^, H3K9me2^low^, HES1^high^ QCCs (red dots) and other cancer cells (blue dots) as 2D digital tumor maps of whole sections from TNBC and other breast tumors based on Cartesian coordinates within each section (Fig. 2a, b, c). For clarity, areas of stromal infiltration, necrosis, or poor image quality were excluded from these maps. Initial inspection of these 2D maps suggested that QCCs displayed a high degree of spatial heterogeneity. Our tumor map approach also enabled us to determine the topographical arrangement of QCCs by analyzing sequential sections from tumors. Figure 3a shows digital tumor maps of five sequential but non-contiguous sections from a representative, untreated, TNBC tumor (control tumor 3), arranged in a 3D stack according to the orientation of each within the primary tumor block. In this particular specimen, QCCs were found in the periphery of some sequential sections (black arrows, Fig. 3a) but not others (white arrows, Fig. 3a).

To ask whether QCCs were enriched in specific regions of a given tumor, we defined QCC-P as the proportion of QCCs in the overall cancer population per section. We also defined QCC-D as the QCC-P per × 20 FOV. We noted a tremendous variance in QCC-D within each section (box and whiskers plot), but found that QCC-P (red bars) was relatively consistent across sections and between tumors (Fig. 3b). Furthermore, QCC-D was not directly proportional to the total cancer cell density (Additional file 3: S3F). This heterogeneity in QCC location and variation in QCC density suggested that QCC topography might not be determined solely by cancer-cell-intrinsic cues, consistent with prior experimental findings that QCCs arise through irregular interaction between cancer cell surface integrin-β1 receptor and extracellular matrix proteins [10, 11].

The heterogeneous localization of QCCs likely was not a technical artifact, because the identification of QCCs required high HES1 signal concurrently with low AKT and H3K9me2 signal, which would not occur due to irregularities in antibody penetration or antigen retrieval alone. In addition, careful study of all tumors did not reveal consistent patterns in the geographical localization of QCCs.

To test whether the variation in QCC percentage and QCC geography between specimens was due to batch-to-batch staining variability, we examined QCC-P, QCC-D, and QCC geography in sequential sections from control tumors 1, 2 and 3 (stained asynchronously) and sequential sections from tumor 4 (stained synchronously) (Figs. 3a and b). We found that QCC percentage (red bars, Fig. 3b) was more similar between contiguous sections (control tumors 1, 2, 4, Fig. 3b) than non-contiguous sections (control tumor 3, Fig. 3b), regardless of synchronous (control tumor 4) or asynchronous (control tumors 1, 2, 3) staining. This suggested that variation in QCC-P between sections more likely reflected biological variability in QCC location within the tumor rather technical variation in staining. In addition, QCCs appeared to be concentrated in geographic regions that extended across multiple sequential sections stained asynchronously (control tumor 3, black arrows, Fig. 3a). The identification of QCCs in similar regions of sequential sections stained asynchronously would be unlikely to happen in the absence of consistent QCC labeling and identification by the TSA-IF system.

### QCCs cluster within primary breast tumors

**Fig. 4:**
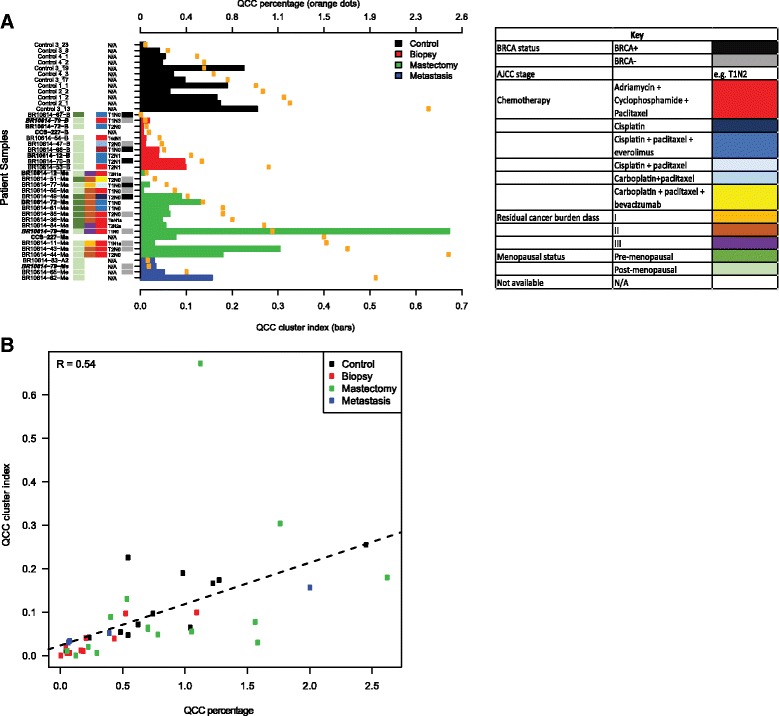
AKT1^low^ quiescent cancer cells (*QCC*) cluster in primary triple negative breast cancer (TNBC) tumor sections and the likelihood of clustering (QCC-cluster index (QCC-CI)) correlates with the QCC percentage (QCC-P). **a** QCC-CI for control samples (*n* = 4, *black bars*), pre-neoadjuvant chemotherapy (NACT) TNBC primary tumor biopsy samples (*n* = 10, *red bars*), post-NACT TNBC primary tumor mastectomy samples (*n* = 15; 1 sample was excluded due to insufficient QCCs to determine QCC-CI, *green bars*) and metastasis samples (*n* = 4, *blue bars*). QCC-P for each patient sample (based on QCC-P of one section per sample) is shown by *orange dots*. Clinical and pathologic data for each patient is shown adjacent to each sample. Each patient is identified by a unique identifier with sample type designated by the letter suffix e.g. *patient BR10614-79* has a biopsy (*BR10614-79-B*), post-NACT mastectomy sample (*BR10614-79-Ma*) and metastatic sample (*BR10614-79-Me*) available. Matched biopsy and mastectomy pairs with residual disease after NACT are labeled *bold*. Biopsy samples with matched mastectomy samples that underwent pCR are *underlined*. A patient with matched biopsy, mastectomy and metastasis samples is identified by *bold italics*. **b** QCC-CI correlates with QCC-P for control, biopsy, mastectomy and metastasis sections (*R* = 0.54)

Visual inspection of this non-random distribution of QCCs suggested that QCCs may actually cluster with other QCCs in untreated primary breast tumors. To further test this hypothesis, we asked if a given QCC was more likely to be found in proximity to other QCCs than to other cancer cells within tumor sections. We defined the QCC-CI as the probability that the nearest neighbors of QCCs were QCCs, rather than other cancer cells (*n* = 100). We plotted the QCC-CI (bars) and QCC-P (orange dots) for different tumor samples, ordered by increasing QCC-P (Fig. 4a). Absent bars indicate samples for which permutation testing was not significant.

Indeed, we found that QCCs were statistically more likely to be found in proximity to other QCCs than other tumor cells in all control primary tumors (black bars, Fig. 4a). Furthermore, QCC-CI positively correlated with QCC-P (*R* = 0.54) across all samples, suggesting that even as QCC numbers increase they remain in close proximity to other QCCs (Fig. 4b). QCC-CI did not directly correlate with total cell numbers in each section, however, suggesting that this observed clustering was not simply a function of increasing tumor cellularity (Additional file 3: S3E).

We then used the QCC identification platform to analyze biopsy (*n* = 10) and mastectomy (*n* = 16) samples from a retrospective cohort of patients with primary TNBC that had been treated with NACT, to test the hypothesis that QCCs in primary tumors persist after NACT.

### QCC clusters persist after chemotherapy in primary and metastatic TNBC tumors

We compared QCC-P (orange dots) and QCC-CI (pre-treatment: red bars; post-treatment: green bars) in primary TNBC tumor samples before and after multi-agent, multi-cycle neoadjuvant chemotherapy (Fig. 4a). We found that QCC-P and QCC-CI were positively correlated in pre-treatment biopsy samples (*R* = 0.89, Additional file 3: S3B), and post-treatment mastectomy samples (*R* = 0.37, Additional file 3: S3C), a finding that was consistent with control samples (*R* = 0.74, Additional file 3: S3A). This suggested that the relationship between QCC clustering and QCC percentage was preserved within primary tumors after chemotherapy. QCC-CI did not correlate with total number of tumor cells, however, further supporting the idea that QCC clustering was not a function of the overall cellularity of the specimen after chemotherapy (Additional file 3: S3E). QCC-CI and QCC-P did not correlate with other clinico-pathologic characteristics such as patient age, menopausal status, BRCA status, tumor stage, pathologic response (Additional file 4: S4), or type of chemotherapy (Fig. 4a).

**Fig. 5:**
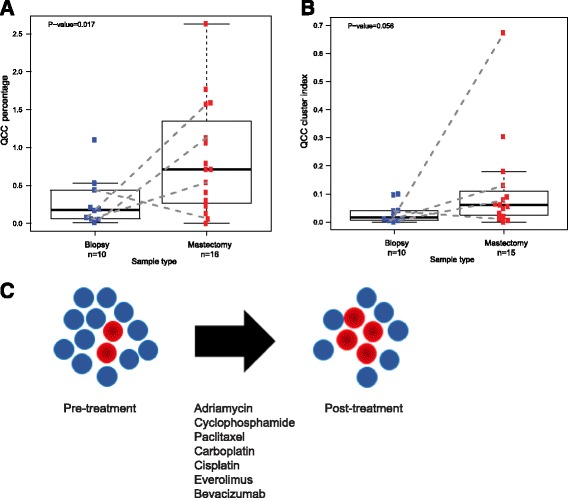
AKT1^low^ quiescent cancer cells (*QCC*s) in primary tumors from patients with triple negative breast cancer persist after multi-agent, multi-cycle neoadjuvant chemotherapy (NACT). **a** QCC percentage appeared to increase in primary breast tumor tissue after NACT (*p* = 0.017) Paired samples are shown by *dotted lines*. **b** QCC cluster index is preserved in primary breast tumor tissue after NACT (*p* = 0.056). **c** Graphical summary of **a** and **b** showing that QCC percentage and clustering persist in post-treatment primary breast tumors after treatment with anthracycline, taxane, platinum-based chemotherapy, and mTOR inhibitors and VEGF inhibitors (*red dots*, QCCs; *blue dots*, non-QCCs)

Significantly, QCC-P appeared to increase after NACT in post-treatment mastectomy specimens compared to pre-treatment biopsies (Fig. 5a). Paired samples are shown by dotted lines (Fig. 5a). This observation suggested that after NACT, QCCs may comprise a larger proportion of residual cells in the tumor, as compared to pre-treatment tumor tissue, as illustrated in Fig. 5c. However, given the limited sampling intrinsic to core biopsy specimens, this finding will require further validation in larger cohorts. QCC-CI was preserved after NACT in post-treatment mastectomy specimens compared to pre-treatment biopsies (Fig. 5b). These data further suggested that QCCs persist as clusters after NACT (Fig. 5c). The increase in QCC-P was consistent with either the preferential survival of QCC clusters post-therapy (i.e. selection) or induction of the QCC state by chemotherapy, but further studies are required to distinguish between these two possibilities.

Finally, we asked whether QCC clusters also exist at metastatic sites after treatment. We determined the QCC-P and QCC-CI in four metastatic TNBC specimens (Fig. 4a, blue bars). As in primary tumors, QCC-P and QCC-CI in metastatic tumors were positively correlated (*R* = 1, Additional file 3: S3D). QCCs were also found in TNBC deposits within axillary lymph nodes (BR10614-62, BR10614-79), brain (BR10614-65-Me) and skin (BR10614-83-Me). Intriguingly, the QCC-CI and QCC-P from a single patient with residual axillary disease after NACT (BR10614-62) also appeared to be significantly greater than that of other sites. These limited but provocative data were consistent with QCC clusters existing not only within primary but also within metastatic tumors.

## Discussion

AKT1^low^ quiescent cancer cells have varied cellular properties that are associated with chemotherapy resistance in experimental models [10–13]. A previous study in tumor samples from five breast cancer patients suggesting that QCCs might survive NACT within human tumors was limited by the use of manual microscopy and the small sample size [11]. Here, we developed a quantitative immunofluorescence microscopy and computational analysis platform to identify and characterize QCCs in clinical tumor specimens, and applied this platform to study a larger cohort of TBNC patients treated with NACT.

For this and future tissue-based studies using quantitative immunofluorescence, we defined QCCs as those cancer cells concurrently expressing the 75^th^ percentile of HES1 fluorescence intensity and the 25^th^ percentile of pan-AKT and H3K9me2 fluorescence intensity, respectively, in that sample. We found that QCCs cluster in highly heterogeneous patterns within pre-treatment primary tumors. The non-random distribution of QCCs is consistent with our prior experimental evidence that micro-environmental effects may determine the QCC state, although additional work is necessary to fully explore this idea in human tumors. Given the degree of QCC heterogeneity, future evaluation of multiple sequential contiguous sections from matched biopsies and untreated whole tumor sections will be needed to define the minimum number of sections necessary to accurately represent the QCC-P for an entire tumor.

QCC clusters also appear to persist after multi-agent, multi-cycle chemotherapy in primary tumors and synchronous and metachronous nodal and distant metastases. Patients in this cohort varied in age, menopausal status, initial disease stage, and BRCA status. These randomly selected patients were also treated with anthracyclines, taxanes, platinum-based therapy, and mTOR-inhibitors and VEGF-inhibitors. Together, these data are consistent with QCCs potentially representing a non-genetically determined cell state that persists after combination chemotherapy of various types at both primary and metastatic sites.

The use of pre-specified fluorescence thresholds to identify QCCs eliminated bias in cell identification and allowed us to quantify QCC-P in patient tumor samples. In addition, representing QCCs and other cancer cells as digital tumor maps allowed us to define the spatial relationships between QCCs and QCC clustering (QCC-CI). This automated approach yielded striking information on the geographical location, density, clustering, and heterogeneity of QCCs not readily apparent with the use of standard, indirect immunofluorescence and manual microscopy. Given the modest numbers and retrospective nature of our cohort, it was not possible to assess the association between QCC-P and/or QCC-CI and patient outcomes such as time to recurrence or overall survival, but future studies will address these important issues. In addition, multiplexing of additional markers should enable further study of the relationship between QCC clusters and other cancer and stromal cell subpopulations within breast and other tumors both before and after treatment.

## Conclusions

The mechanisms that allow TNBC tumors to survive neoadjuvant chemotherapy are incompletely understood. Evidence suggests that proliferative heterogeneity may contribute to primary chemotherapy resistance in patients with TNBC. However, the identification of a putative quiescent, drug-resistant, cancer cell state in residual TNBC after NACT has remained elusive. AKT1^low^ quiescent cancer cells are a quiescent, epigenetically plastic, and chemotherapy-resistant subpopulation initially identified in experimental cancer models. Here, we reproducibly demonstrated the presence of QCCs in primary and metastatic human breast tumors using automated, quantitative, immunofluorescence microscopy coupled with computational and statistical analysis. We showed that QCCs exist as non-random and heterogeneously distributed clusters within primary tumors. In addition, these QCC clusters persist after treatment with multi-agent, multi-cycle, NACT in both residual primary tumors and nodal and distant metastases in patients with TNBC. Together, these data potentially qualify QCCs as a non-genetic cell state that persists after NACT in TNBC patients, and warrants further study.

## Additional files

Additional file 1: **S2** Antibody target specificity is unaffected by sequence of primary antibody application. Merged (*right*) and single color (*left*) confocal microscopy images at × 60 of an untreated primary TNBC tumor stained in an alternate sequence: pan-AKT ➔ H3K9me2 ➔ HES1 (c.f. standard sequence of H3K9me2 ➔ pan-AKT ➔ HES1) demonstrating consistent cytoplasmic pan-AKT (*green*) and HES1 (*red*) staining and nuclear H3K9me2 (*yellow*) staining in an example QCC (*white arrows*). (PDF 3624 kb)

Additional file 2: **S1** Determination of fluorescence intensity thresholds and staining reproducibility. For each marker (HES1, H3K9me2, pan-AKT) the proportion of cells at a specific fluorescence intensity level was different between sequential sections from control tumor 4, stained simultaneously (S1A and S1B, respectively). QCC percentage (*red bars*) and QCC density (*box and whisker plots*) in control tumors 1–4 increased proportionally at 25%, 33%, and 50% thresholds (S1C, S1D, and S1E, respectively). (PDF 2666 kb)

Additional file 3: **S3** QCC-P and QCC-CI are positively correlated in control samples and in biopsy and mastectomy samples after neoadjuvant chemotherapy. Correlation of QCC-CI and QCC-P in control samples (**A**), pre-treatment biopsy samples (**B**), post-treatment mastectomy samples (**C**), and metastatic samples (**D**). **E** QCC-CI does not correlate with total number of cancer cells in each sample. **F** QCC density (QCC-P per × 20 field of view) in control samples does not positively correlate with cancer cell density (cancer cells per × 20 field of view). (PDF 3045 kb)

Additional file 4: **S4** QCC-P of pre-treatment biopsies and matched mastectomy specimens with pathologic complete response after NACT is not significantly different from QCC-P of pre-treatment biopsies and matched mastectomy specimens with residual disease after NACT. Plot shows QCC percentage of pre-treatment biopsies (*n* = 8, *blue dots*) ordered by the pathologic response of their matched post-treatment mastectomy specimens (residual disease vs. pathologic complete response) *p* = 0.39. (PDF 731 kb)

## Abbreviations

DAPI: 4',6-Diamidino-2-phenylindole
ER: Estrogen receptor
FFPE: Formalin-fixed paraffin-embedded
FITC: Fluorescein isothiocyanate
FOV: Field of view
H&E: Hematoxylin and eosin
HER2: Human epidermal growth factor receptor 2
ITGB1: Integrin β1
NACT: Neoadjuvant chemotherapy
pCR: Pathologic complete response
QCC: AKT1^low^ quiescent cancer cells
QCC-CI: AKT1^low^ quiescent cancer cell cluster index
QCC-D: AKT1^low^ quiescent cancer cell density
QCC-P: AKT1^low^ quiescent cancer cell percentage
RCB: Residual cancer burden
TNBC: Triple negative breast cancer
TSA: Tyramide-signal-amplified
