# Supplementary figures and images for: AKT1low quiescent cancer cells persist after neoadjuvant chemotherapy in triple negative breast cancer
Source: Breast Cancer Res. 2017 Aug 1;19:88. doi: 10.1186/s13058-017-0877-7 (PMC5540189; doi:10.1186/s13058-017-0877-7)

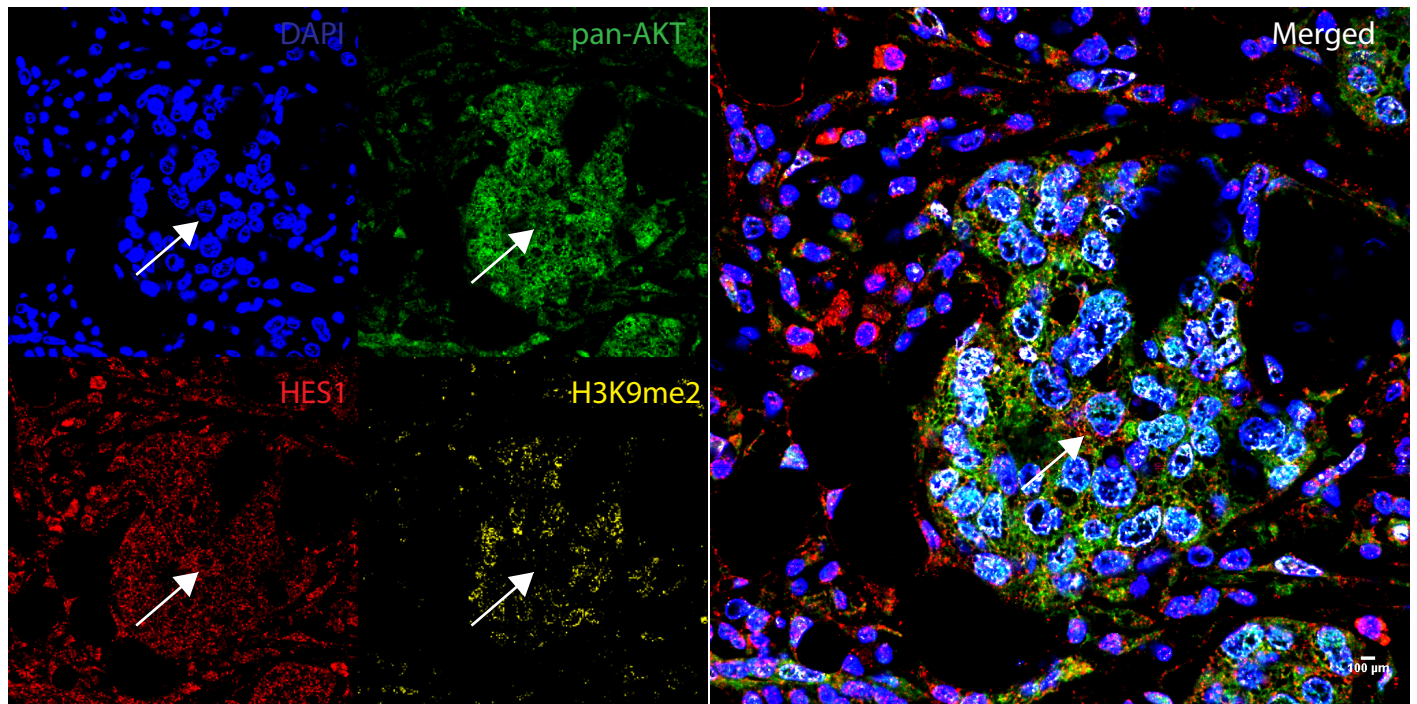

Supplement: Supplementary file 1 — S2 Antibody target specificity is unaffected by sequence of primary antibody application. Merged (right) and single color (left) confocal microscopy images at × 60 of an untreated primary TNBC tumor stained in an alternate sequence: pan-AKT ➔ H3K9me2 ➔ HES1 (c.f. standard sequence of H3K9me2 ➔ pan-AKT ➔ HES1) demonstrating consistent cytoplasmic pan-AKT (green) and HES1 (red) staining and nuclear H3K9me2 (yellow) staining in an example QCC (white arrows). (PDF 3624 kb) [file 13058_2017_877_MOESM1_ESM.pdf]

A

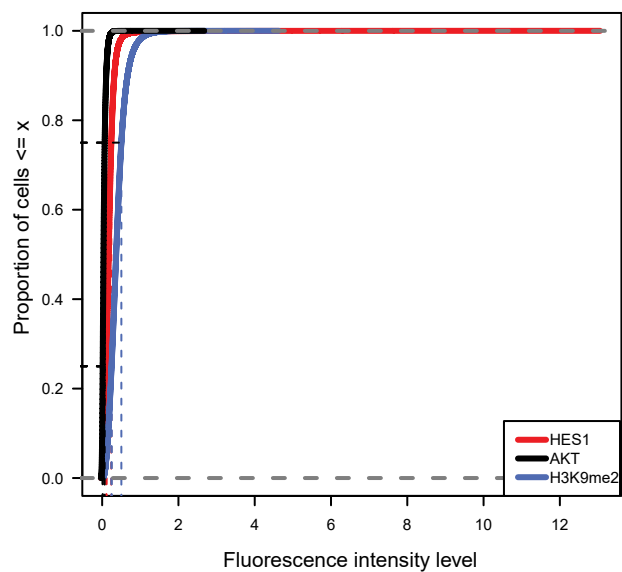

B

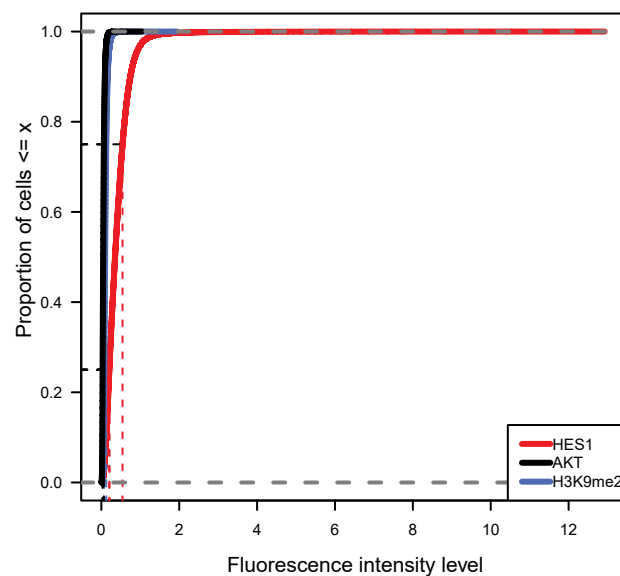

C

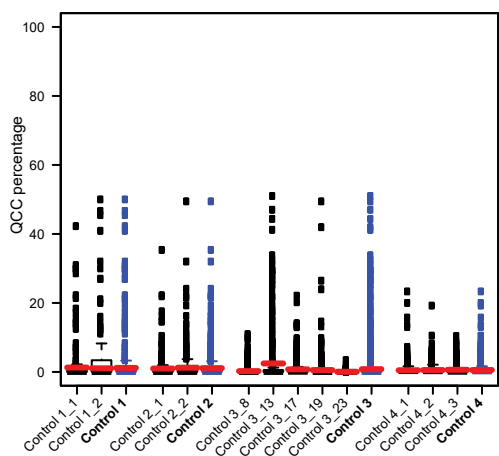

D

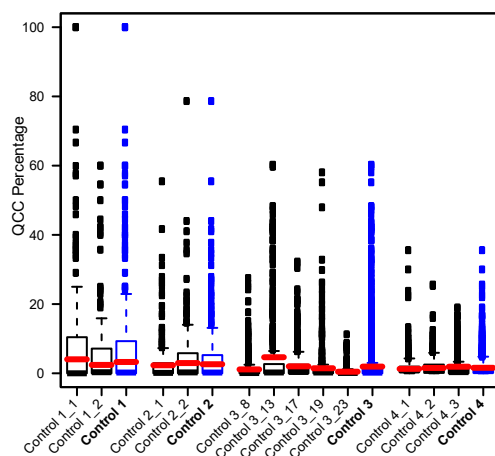

E

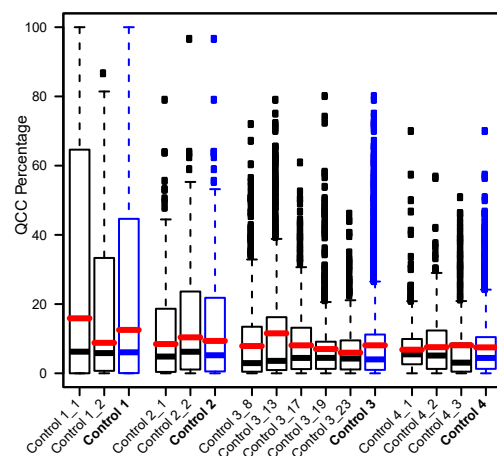

F

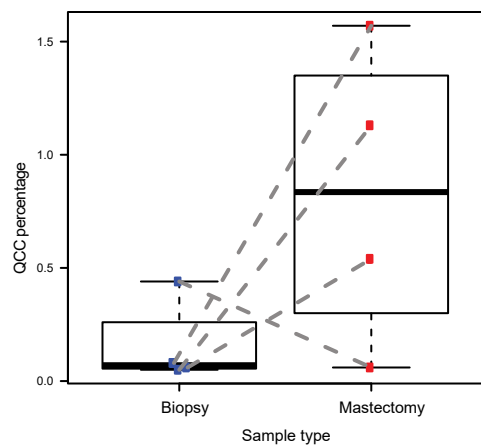

G

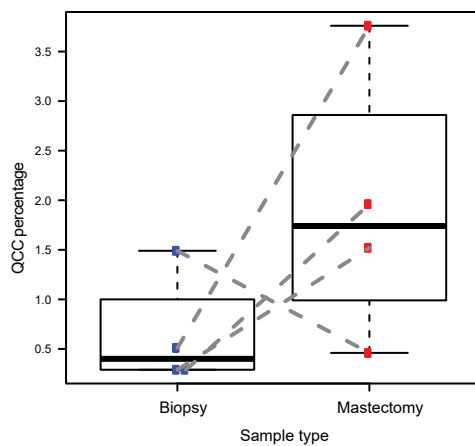

H

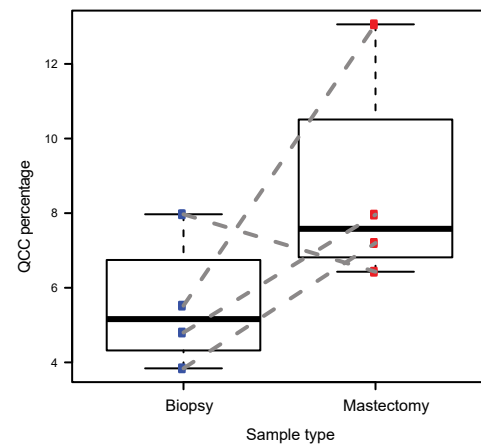

Supplement: Supplementary file 2 — S1 Determination of fluorescence intensity thresholds and staining reproducibility. For each marker (HES1, H3K9me2, pan-AKT) the proportion of cells at a specific fluorescence intensity level was different between sequential sections from control tumor 4, stained simultaneously (S1A and S1B, respectively). QCC percentage (red bars) and QCC density (box and whisker plots) in control tumors 1–4 increased proportionally at 25%, 33%, and 50% thresholds (S1C, S1D, and S1E, respectively). (PDF 2666 kb) [file 13058_2017_877_MOESM2_ESM.pdf]

A

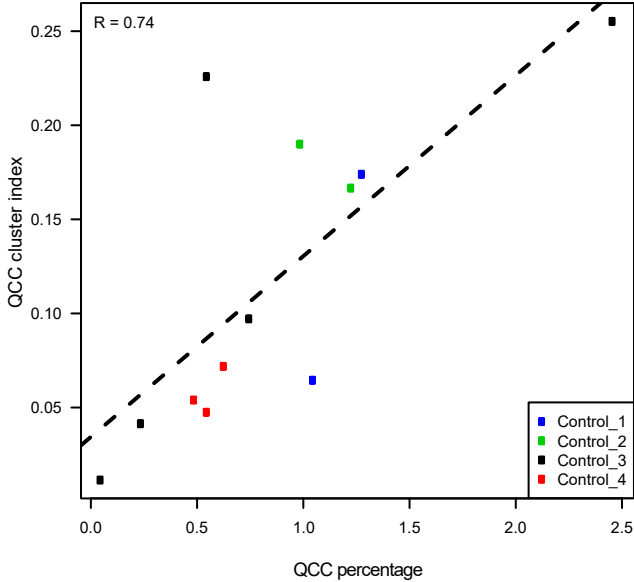

B

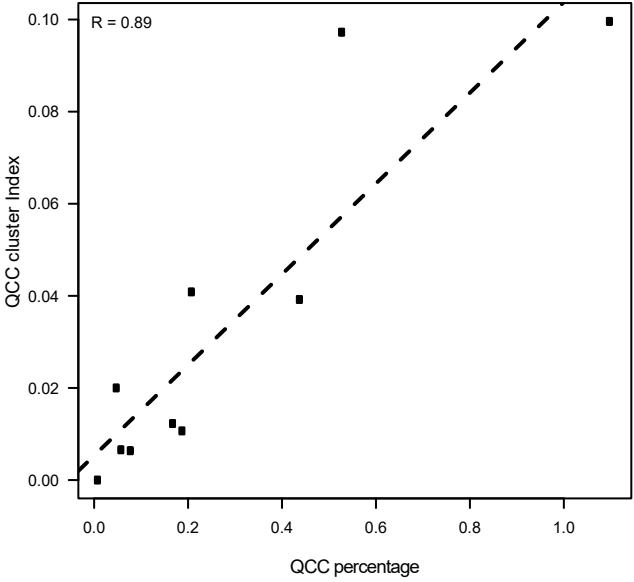

C

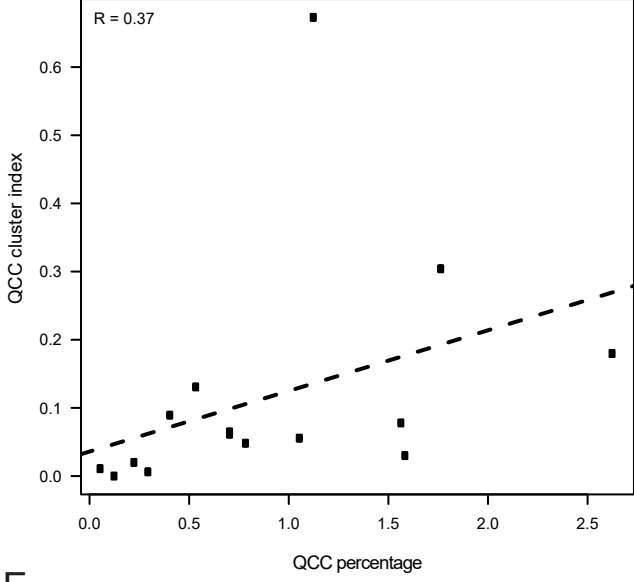

D

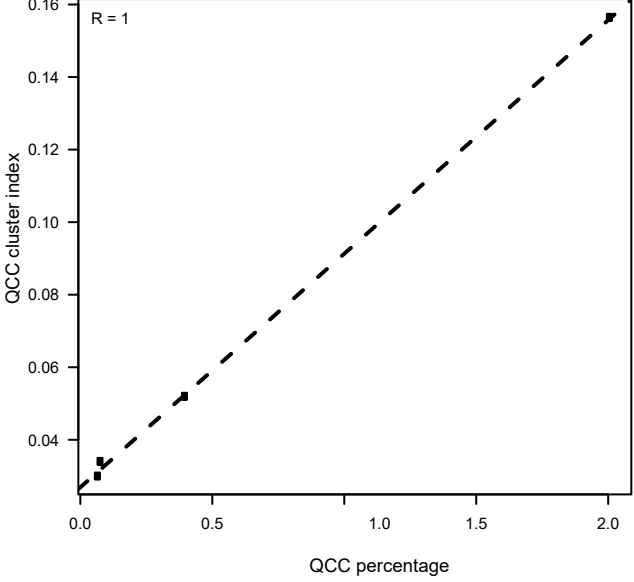

E

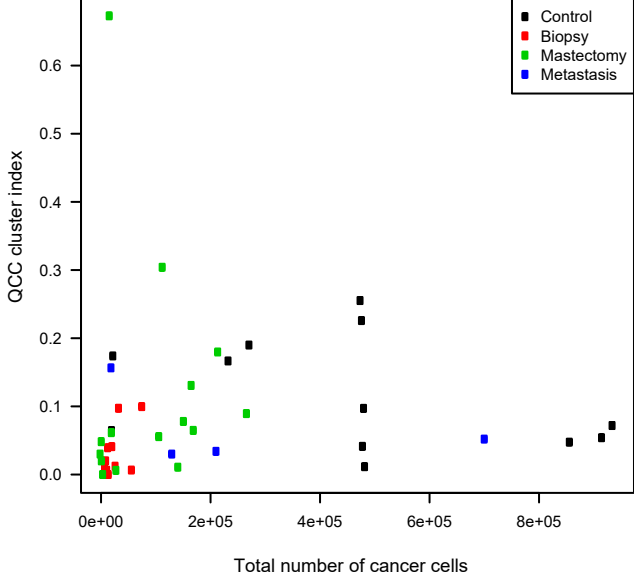

F

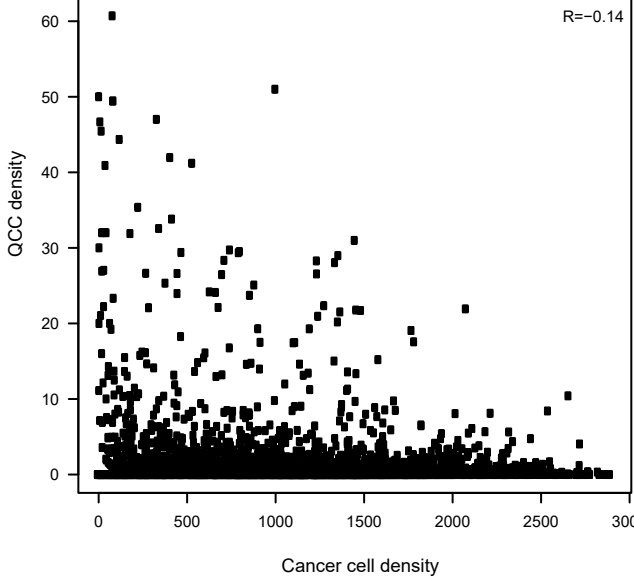

Supplement: Supplementary file 3 — S3 QCC-P and QCC-CI are positively correlated in control samples and in biopsy and mastectomy samples after neoadjuvant chemotherapy. Correlation of QCC-CI and QCC-P in control samples (A), pre-treatment biopsy samples (B), post-treatment mastectomy samples (C), and metastatic samples (D). E QCC-CI does not correlate with total number of cancer cells in each sample. F QCC density (QCC-P per × 20 field of view) in control samples does not positively correlate with cancer cell density (cancer cells per × 20 field of view). (PDF 3045 kb) [file 13058_2017_877_MOESM3_ESM.pdf]

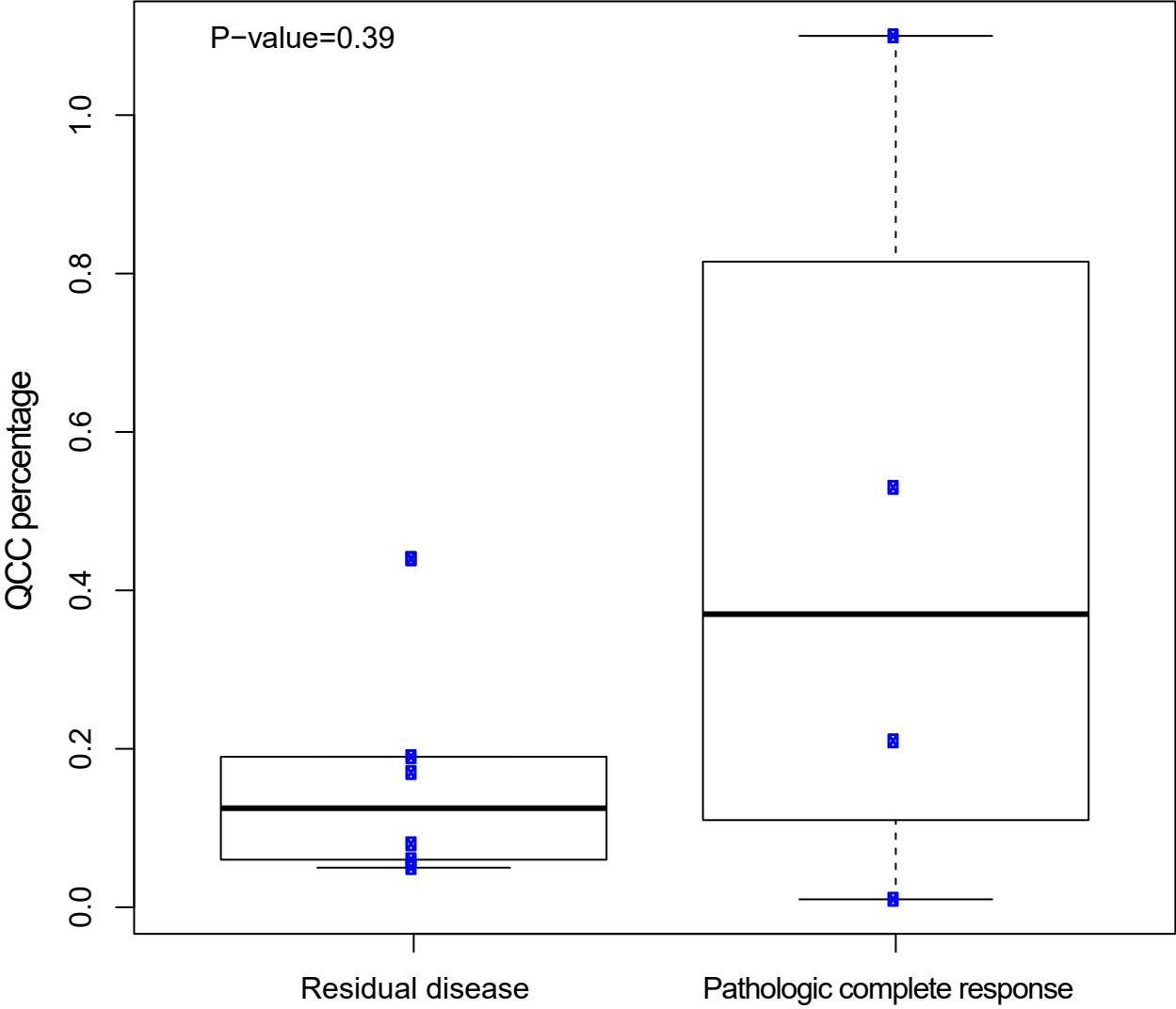

Supplement: Supplementary file 4 — S4 QCC-P of pre-treatment biopsies and matched mastectomy specimens with pathologic complete response after NACT is not significantly different from QCC-P of pre-treatment biopsies and matched mastectomy specimens with residual disease after NACT. Plot shows QCC percentage of pre-treatment biopsies (n = 8, blue dots) ordered by the pathologic response of their matched post-treatment mastectomy specimens (residual disease vs. pathologic complete response) p = 0.39. (PDF 731 kb) [file 13058_2017_877_MOESM4_ESM.pdf]
